# Ergonomic risk management process for safety and health at work

**DOI:** 10.3389/fpubh.2023.1253141

**Published:** 2023-11-09

**Authors:** Oleg Bazaluk, Vitaliy Tsopa, Serhii Cheberiachko, Oleg Deryugin, Dmytro Radchuk, Oleksandr Borovytskyi, Vasyl Lozynskyi

**Affiliations:** ^1^Belt and Road Initiative Center for Chinese-European Studies (BRICCES), Guangdong University of Petrochemical Technology, Maoming, China; ^2^Department of Management and Economics, International Institute of Management, Kyiv, Ukraine; ^3^Department of Labour Protection and Civil Safety, Dnipro University of Technology, Dnipro, Ukraine; ^4^Department of Transportation Management, Dnipro University of Technology, Dnipro, Ukraine; ^5^Department of Mining Engineering and Education, Dnipro University of Technology, Dnipro, Ukraine

**Keywords:** ergonomics, risk, safety, dangerous factors, health, disease, consequences

## Abstract

**Purpose:**

The paper aims to provide the main principles and practical aspects of the model, to present the process of identifying, determining the level, as well as assessing and managing occupational and ergonomic risks.

**Methods:**

To conduct the research, as well as to identify the influence of various dangerous factors related to the working posture, pace, rhythm of work performance, equipment and individual characteristics of the employee’s health condition, methods of complex analysis and synthesis, formal and dialectical logic are used to study the essence of the concept of occupational and ergonomic risks. Additionally, induction and deduction methods are used to examine the cause-and-effect relationships between dangers, dangerous factors, dangerous event, and the severity of consequences to determine the level of occupational and ergonomic risks based on the improved bow-tie model. The proposed approach effectiveness is tested based on the assessment of occupational and ergonomic risks of forest workers (loggers) with the participation of five experts to identify dangerous factors and develop precautionary measures.

**Results:**

An algorithm for managing occupational and ergonomic risks has been developed, consisting of eleven steps, which can be divided into three steps: preparatory, main and documented. It has been determined that occupational and ergonomic risk is the probability of a dangerous event occurring due to employee’s physical overload and its impact on the severity of damage to the employee’s physical health. The level of occupational and ergonomic risk management is determined taking into account the probability (frequency), intensity and duration of physical overload, as well as the employee’s adaptation index to physical overload and his/her health index.

**Conclusion:**

The novelty is the substantiation of the principles of occupational and ergonomic risk management, which are based on the bow-tie model and predict the impact on the probability and severity of consequences of a dangerous event, taking into account dangerous factors. Forms for drawing up occupational and ergonomic risk maps have been developed, in which it is necessary to consider interaction of occupational hazards and occupational-ergonomic risk – physical overload.

## 1. Introduction

Occupational safety and health protection is a priority area for development. New approaches are being developed and production processes and models are being updated to improve the effectiveness of occupational health and safety management systems. However, in the area of occupational safety and health, greater attention has been given to finding ways to reduce losses within organizations, leading to a constant conflict over the distribution of funding between different systems. A more promising field of activity is provided by ergonomics, which, according to clause 2.21 of ISO 26800:2011 standard, “is a scientific discipline that studies the interaction of a person and other system elements (3.5), as well as the field of activity on application of the theory, principles, data and methods of this science to ensure human well-being and optimize the whole system performance.” Thus, this allows specialists to reduce the impact of dangers and dangerous factors to increase labor productivity, which is more attractive to employers in terms of financial profits (1, 2). On the other hand, business owners face significant financial losses due to diseases or injuries to the musculoskeletal system of their employees (3). Therefore, there is a need to identify ergonomic risks and develop appropriate recommendations to preserve the health of employees (4–6).

The main factors of interest for ergonomics, as well as for occupational safety, include hygienic, anthropometric, physiological, psychophysiological and psychological factors that cause deterioration in the physical and mental health of employees (7). A significant number of them, due to limited financial and material resources in the organizations, require the introduction of a process for managing occupational and ergonomic risks, the purpose of which, inseparable from occupational safety, is not only to reduce injuries and occupational morbidity, but also the creation and protection of values, the main of which are the life and health of an employee.

**Figure 1:**
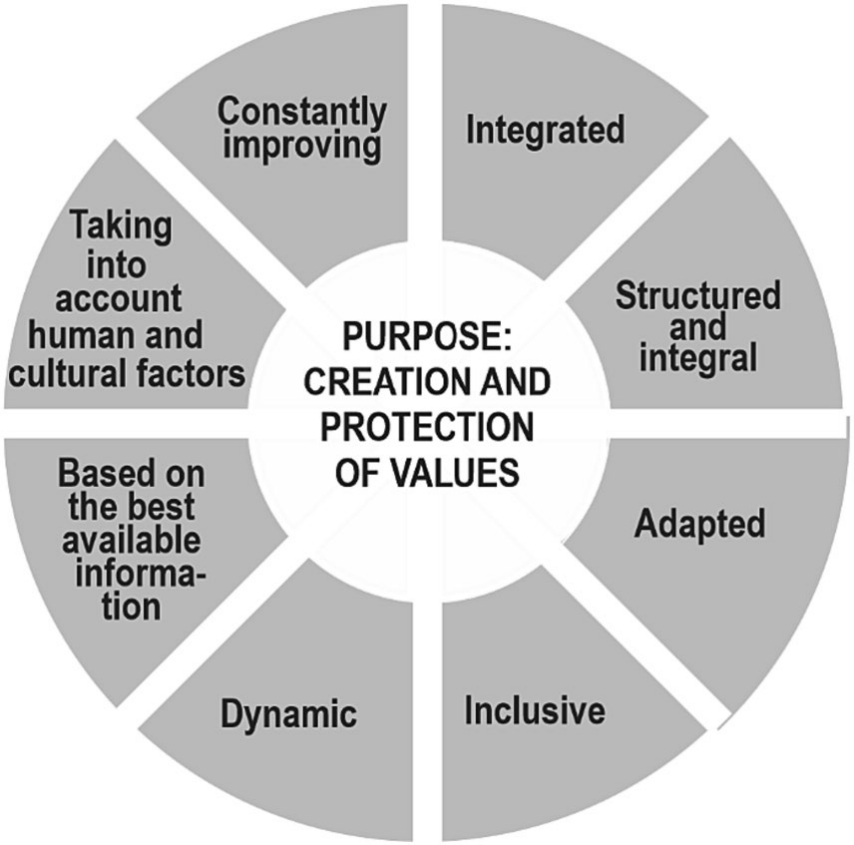
Risk management principles.

To successfully implement risk management at the enterprise, it is necessary to rely first of all, on ISO 31000:2018 standard (the National Standard of Ukraine ISO 31000:2018 “Risk management. Principles and guidelines”), which defines the basic principles and essence of the risk management process (Figure 1) (8). The creation of algorithms for managing occupational and ergonomic risks of an organization is an urgent task, the solution of which will improve the effectiveness of occupational health and safety management systems, as well as stimulate innovation and contribute to the achievement of goals to reduce injuries and occupational diseases.

In order to prevent the development of the Musculoskeletal System Diseases (hereinafter – MSDs), more and more attention is paid to the assessment of occupational and ergonomic risks, as well as various factors that worsen working postures when performing production tasks. Many different methods are used for this purpose, which can be divided into three main groups: subjective judgment, systematic observation and direct observation (9–12). Each of them has its own advantages. For example, subjective judgment methods allow you to quickly assess a situation and make a decision within a limited time frame. The following methods use special checklists, such as the “RULA/REBA” method (13, 14), which are based on assessing the scores, taking into account the difficulty of uncomfortable postures when performing certain production operations. Thus, the specified methods make it possible to quantify various indicators associated with an employee’s working posture (15, 16), comparison with which allows the evaluator to set the appropriate scores. At the same time, the total number of these scores does not allow determining the risk of an occupational disease or injury, since it evaluates only one of the ergonomic risk (ER) components: a load index comparable to the probability of occurrence of a dangerous event. However, the REBA or RULA approach lacks a second necessary component for risk assessment: the severity of consequences, which is the main disadvantage of these approaches. In addition, they also do not take into account the employee’s individual health. It is precisely the need to assess its influence that is discussed in the following works (17, 18). The authors insist that when studying ER, it is necessary to consider the compatibility of production conditions with the physiological, psychological and anthropometric properties of employees with an assessment of their health (19, 20). The need to take into account individual health when assessing ER is discussed in some publications (21, 22).

The purpose of the research is to develop the principle for managing occupational and ergonomic risks in the employee’s workplace when performing professional activities.

## 2. Methods

According to the European Agency for Health and Safety at Work definition of ergonomic risk (23): ergonomic risk is a risk caused by physical overload, repetitive movements or unnatural postures during work, which can lead to fatigue, mistakes, accidents, occupational diseases or musculoskeletal system disorders. Ergonomic risks are complex and multidimensional by their nature, they can affect the loss of employee’s productivity, his/her physical and psychological health. If they occur in the workplace, they can directly cause or worsen a current health condition. Different types of movements have very different impacts on occupational MSDs, and there are important factors, such as age, health condition and gender of the employee that need to be considered when managing ergonomic risks.

The general ergonomic risk is a combined risk and it consists of four possible variants:

- Occupational and ergonomic risk of a dangerous event, incident or accident;
- Occupational and ergonomic risk of occupational MSDs;
- Psychosocial ergonomic risk;
- Ergonomic risk of work performance.

In this paper, we will consider the purely professional and ergonomic risk of occupational MSDs, taking into account the following significant dangerous factors:

- The direction of repeated movements of the joints, the magnitude of effort, the level of load and the state of activity;
- Working environment at the workplace;
- Equipment, appliances and tools at the workplace;
- Health condition of the employee (age of the employee);
- Gender of the employee.

**Figure 2:**
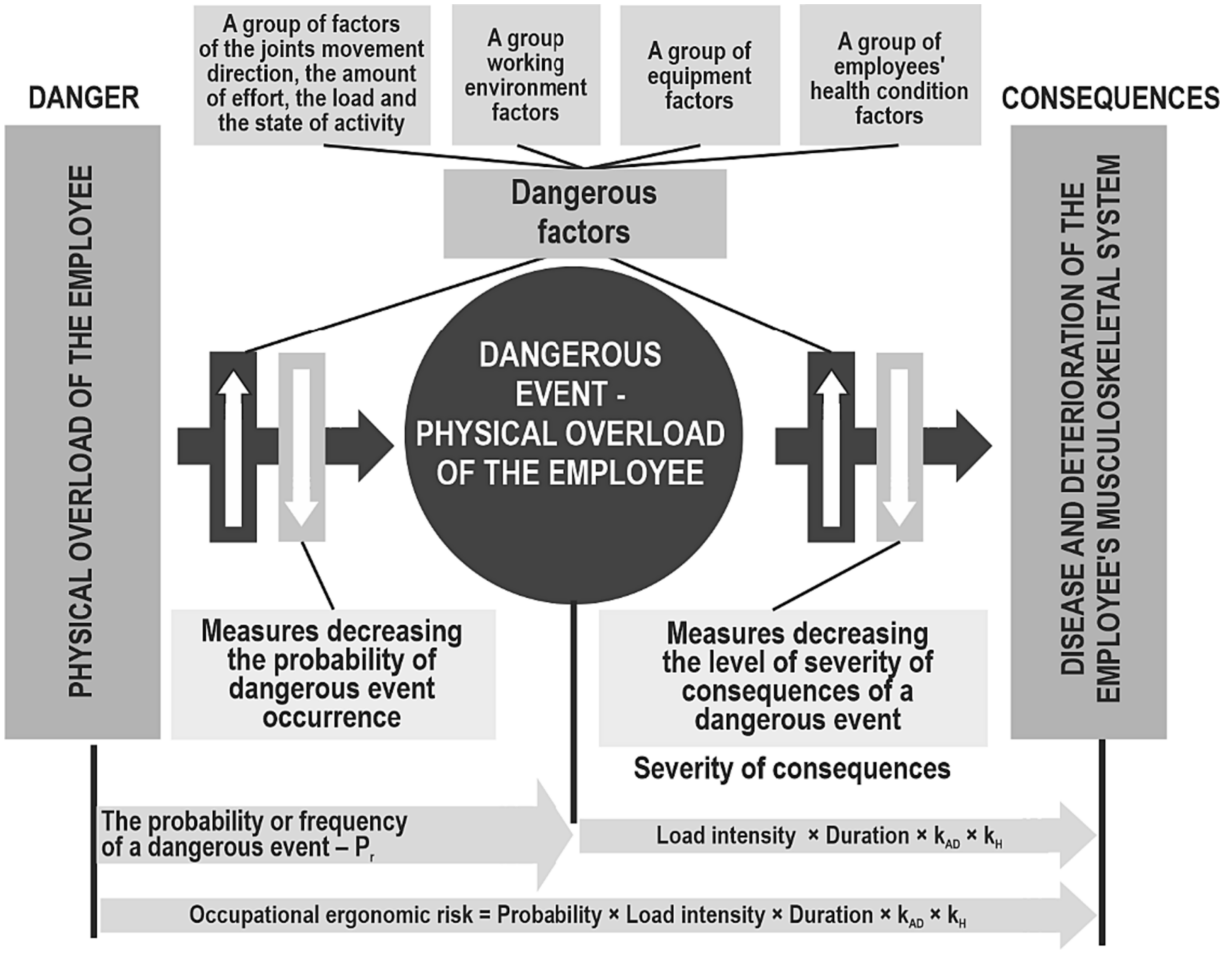
Occupational and ergonomic risk model.

As for the psychosocial ergonomic risk, the danger is posed by musculoskeletal system disease – this means physical overload of the employee, and a dangerous event is a physical exhaustion, while the consequences of which are the occurrence of occupational MSDs. Moreover, the increase in the probability of the dangerous event occurrence – physical overload of employees, as well as the severity of consequences (severity of an occupational disease) is influenced by a number of different dangerous factors associated with working posture, pace, rhythm of work performance, environmental and hygienic factors, equipment and individual characteristics of the employee (7, 24). The level of health condition corresponds to the employee’s adaptive capabilities to tolerate/adapt to inconveniences when performing production tasks without consequences for health. On the other hand, precautionary and protective measures should be organized at each workplace that reduce the influence of dangerous factors, which must be based on the principles of occupational and ergonomic risk management. Given the bow-tie model (25, 26), which is the best way to reveal cause-and-effect relationships between danger, a dangerous event and the severity of consequences, it is possible to obtain an appropriate idea of occupational and ergonomic risks (Figure 2).

Thus, having identified the physical overload of an employee as the main danger, which can lead to a dangerous event – physical overload under the influence of a number of dangerous factors – the main components of the occupational and ergonomic can been determined, namely: the frequency of the dangerous event occurrence by identifying the value of physical overload and the severity of consequences based on the physical health and adaptability of the employee. At the same time, given that the dangerous event occurrence depends on a group of dangerous factors, such as the direction of joint movement, the magnitude of effort and the level of load, the severity of consequences can be expressed through the intensity and duration of the load. From this, a conclusion can be drawn about the degree of the disease severity, as well as the period of its development.

**Figure 3:**
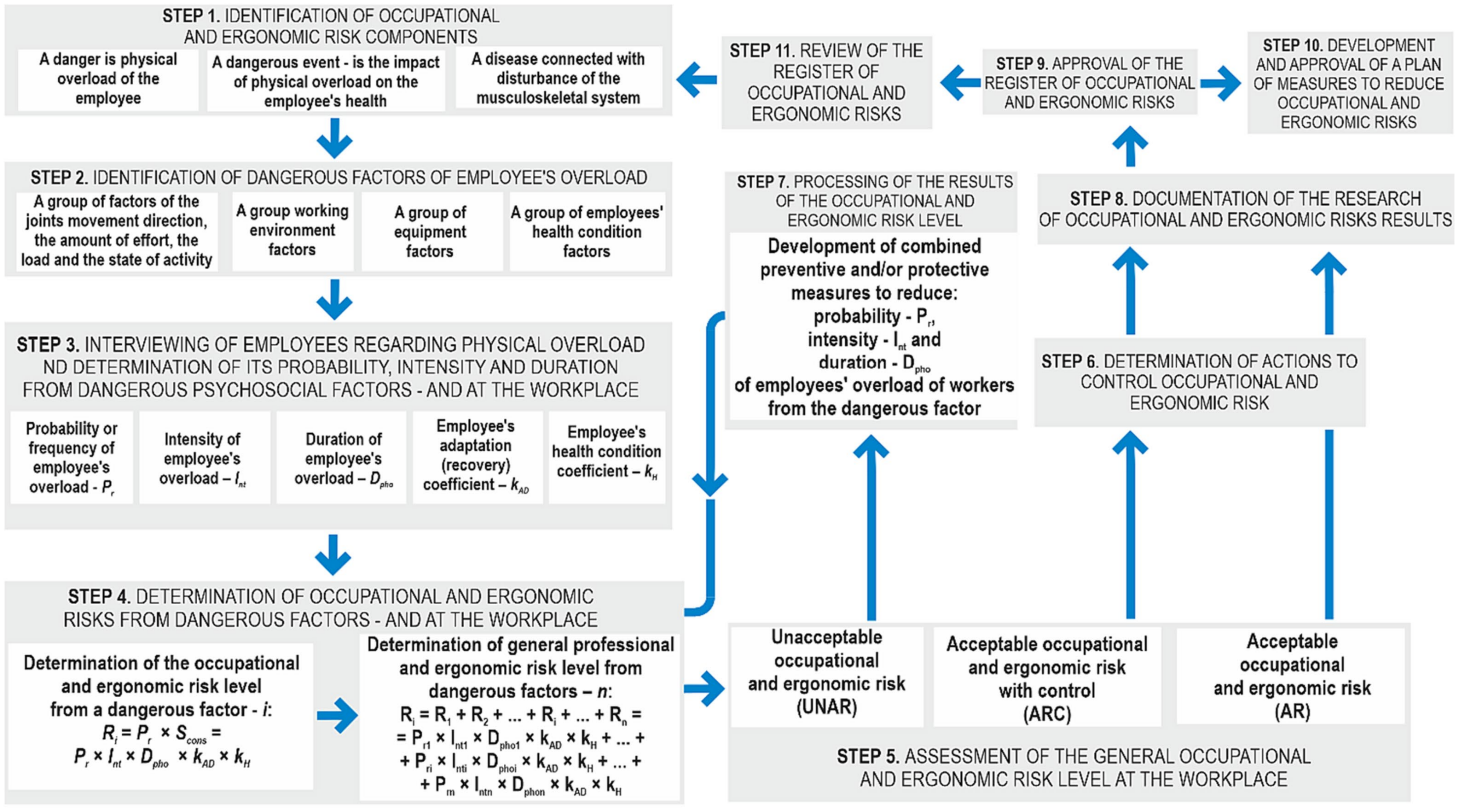
Occupational and ergonomic risk managing process.

The constructed model (Figure 2) makes it possible to develop an appropriate algorithm for the process of managing occupational and ergonomic risks, which consists of eleven steps (Figure 3). It differs from the existing ones by the procedure for determining both the level of occupational and ergonomic risk from a specific dangerous factor, and the overall level of risk (taking into account all dangerous factors). This approach makes it possible to understand, from the total influence of all dangerous factors on the probability of the dangerous event occurrence, only the most important ones that require rapid response.

The first three steps related to identifying the components of occupational and ergonomic risks, dangerous factors causing employee’s overload, and the procedures for their examination belong to the preparatory step, which involves the preparation of documents and process components, in particular:

- Register of dangerous occupational and ergonomic risks.
- Determining the level of occupational and ergonomic risk and its components.
- Scales of probability (frequency) of the occurrence of a dangerous event and scales of severity of the consequences from the dangerous event occurrence and their components of occupational and ergonomic risk.
- Matrices for assessing occupational and ergonomic risks.
- Forms of the maps of occupational and ergonomic risks.
- Forms of the employee’s questionnaire about dangerous occupational and ergonomic factors, as well as components of occupational and ergonomic risk.

We suggest using the form given in the Table to develop a register of dangerous occupational and ergonomic factors. 1. Three groups of dangerous factors are highlighted in the form: (1) direction of joint movement, the magnitude of effort, the level of load and state of activity; (2) working environment; and (3) equipment and infrastructure. The employee’s adaptation to physical overload and his/her health condition is taken into account. For each dangerous factor, it is indicated the possible level of intensity of the employee’s physical overload and its duration, as well as the consequences that the employee’s physical overload leads to.

After the preparatory step, we proceed to the fourth step “Determining the level of occupational ergonomic risk and its components,” which can be conducted using two approaches (See Table 1).

**Table 1:** An example of the register of dangerous occupational and ergonomic factors.

No	Group name of the dangerous psychosocial factors	Dangerous factors	Consequences, MSDs
1	Group of factors in the direction of joint movement, effort magnitude, load level and activity state	1.1 Angle of the head inclination relatively to the body; 1.2 Angle of the torso inclination relatively to the workplace; 1.3 Placement of hands relatively to the body; 1.4 Rhythm and pace of work; 1.5 Dynamic loads.	Muscle pain, muscle strain, ligament rupture, disease development (arthritis, arthrosis, intervertebral hernia, bursitis, etc.), injury
2	Group of dangerous factors – working environment	2.1 Illumination; 2.2 Noise; 2.3 Vibration; 2.4 Air temperature; 2.5 Weather conditions	Deterioration of the employee's physical condition, vision, hearing, tactile sensations, development of vibration disease, manifestations of body overheating, heat stroke
3	Group of dangerous factors – equipment	3.1 Number of repetitive operations; 3.2 Number of objects under the control; 3.3 Equipment weight.	The development of fatigue, which leads to mistakes and injuries, nervous strain

### 2.1. The first approach

Determining the level of occupational and ergonomic risk and its components in the traditional way using the formula:


$$R_{i}=P_{r}\times S_{\mathrm{cons}}\text{,}$$

where, *P_r_* is probability of physical overload; *S_cons_* is severity of the consequences of physical overload.

It is recommended to use the scale of probability (frequency) of the dangerous event occurrence and the scale of severity of the consequences from the dangerous event occurrence, given in the Tables 2 and 3, respectively. The issues of constructing a scale of probability (frequency) of the dangerous event occurrence and a scale of severity of the consequences are presented in detail (27). Having a scale of probability (frequency) of the dangerous event occurrence (Table 2) and a scale of severity of the consequences from the dangerous event occurrence (Table 3), it is possible to construct a matrix for assessing occupational and ergonomic risks (Table 4).

**Table 2:** Frequency scale of the dangerous event occurrence.

No	Frequency level of the dangerous event occurrence	Indication	Frequency criterion for the dangerous event occurrence	Score
1	Extremely high	A	At least 1 time per hour	5
2	High	B	At least once per work shift	4
3	Medium	C	At least once per week	3
4	Low	D	At least once per month	2
5	Absent	E	Absent	1

**Table 3:** Scale of severity of consequences from the dangerous event occurrence.

No	Severity level of the dangerous event consequences	Indication	Criteria for human MSDs	Score
1	Extremely high	I	MSDs, which lead to a complete loss of working capacity, the onset of disability (disability of the 1st group)	5
2	High	II	MSDs, which lead to partial loss of working capacity (disability of the II group)	4
3	Medium	III	Average injury or illness without loss of working capacity, but with long-term treatment – more than three months and less than a year	3
4	Low	IV	Minor injury or illness without disability, with treatment lasting more than three days but less than seven days	2
5	Absent	V	No injuries or illnesses	1

**Table 4:** Assessment matrix of occupational and ergonomic risks.

Assessment matrix of occupational and ergonomic risks			Scale of severity of consequences from the dangerous event occurrence				
			I	II	III	IV	V
			5 scores	4 scores	3 scores	2 scores	1 score
Frequency scale of the dangerous event occurrence	A	5 scores	25	20	15	10	5
	B	4 scores	20	16	12	8	4
	C	3 scores	15	12	9	6	3
	D	2 scores	10	8	6	4	2
	E	1 scores	5	4	3	2	1

In this case, the following risk acceptance criteria are used for risk assessment: unacceptable risk – more than 14 and from 14 to 25; acceptable risk with scores from 8 to 13; acceptable risk without verification is less than 8 and from 1 to 7.

### 2.2. The second approach

Determination of the level of occupational and ergonomic risk, as well as its components, is assumed taking into account the probability of physical/mental overload; the severity of the consequences (determined by the intensity and duration of physical/mental overload); the index of employee’s adaptability to physical/mental overload; employee’s health condition:


$$R_{i}=P_{r}\times P_{pho}=P_{r}\times I_{\mathrm{nt}}\times D_{pho}\times k_{\mathrm{AD}}\times k_{H}\times k_{G}$$

where, *P_r_* – probability of physical overload; *I_nt_* – intensity of physical overload; *k_AD_* – the index of employee’s adaptability (recovery) to physical overload; *D_pho_* – duration of physical overload; *k_H_* – the employee’s health condition index; *k_G_* – the coefficient taking into account the employee’s gender.

We determine the intensity of physical overload (Table 5) and the duration of physical overload (Table 6). To create a scale of physical overload intensity, the following research recommendations are used (28, 29).

**Table 5:** Scale of physical overload intensity.

No	Overload intensity level	Indication	Criterion for physical overload, %	Score
1	Extremely high	a	75–100	5
2	High	b	50–75	4
3	Medium	c	25–50	3
4	Low	d	1–25	2
5	Absent	e	0	1

**Table 6:** Scale of physical overload duration.

No	Overload duration level	Indication	Criterion of physical overload duration	Score
1	Extremely high	1	More than a half of a year	5
2	High	2	Less than a half of a year, but more than a month	4
3	Medium	3	Less than a month, but more than a week	3
4	Low	4	No more than a week	2
5	Absent	5	Absent	1

Based on the scale of physical/mental overload intensity (see Table 5) and the scale of physical overload duration (see Table 6), we construct a matrix for assessing the occupational and ergonomic risk severity depending on the intensity and duration of physical/mental overload in scores (Table 4). The indices for employee’s adaptability (recovery) and the employee’s health condition are determined according to Tables 7 and 8, respectively. To determine the employee’s adaptability (recovery) index, any proposed methods can be used (30–33). For example, for workers performing physical work, the “Map of Adaptation Process” method (34) or the Harvard Step Test (35, 36), based on assessing heart rate or changes in blood pressure, can be used. The procedure consists of testing immediately after performing physical work and certain time after recovery (37). As a rule, this procedure can be carried out during the regular preventive examination of employees. The coefficient of the employee’s physical health condition can be determined from the employee’s medical history.

**Table 7:** Index of the employee’s adaptability (recovery).

No	Employee’s adaptability	Indication	Description	Index
1	Extremely high	*k_AD1_*	The employee is quickly adapted to physical overload and he/she quickly recovers	0.1–0.25
2	High	*k_AD2_*	The employee is not quickly adapted to physical overload and he/she does not recover quickly	0.25–0.50
3	Medium	*I_ad 3_*	It is difficult for the employee to be adapted to physical overload and it is difficult to recover	0.50–0.75
4	Low	*k_AD4_*	The employee does not adapt well to physical overload and he/she does not recover well	0.75–1.00
5	Absent	*k_AD5_*	The employee does not adapt to physical overload and he/she does not recover	1.00

**Table 8:** The index of the employee's physical health condition.

No	Employee’s health condition	Indication	Description	Coefficient
1	Extremely high	*K_H1_*	The employee has no significant health problems and no chronic diseases	0.05–0.1
2	High	*K_H2_*	The employee has health problems and does not have chronic diseases	0.1–0.25
3	Medium	*K_H3_*	The employee has significant health problems and does not have chronic diseases	0.25–0.50
4	Low	*K_H4_*	The employee has poor health and one chronic disease unrelated to MSDs	0.50–0.75
5	Absent	*K_H5_*	The employee is constantly sick, has significant health problems and more than one chronic disease related to MSDs	0.75–1.00

The gender differences should also be taken in to account through the gender index (Table 9), since there is enough research on the difference in strength and power relative to body weight between men and women (38–40).

**Table 9:** Employee’s gender index.

No	Employee’s gender index	Indication	Employee's gender index score
1	Male	*K_Gmaile_*	1.0
2	Female	*K_Gfemale_*	2.0

In the fifth step, to determine the criteria for acceptability and unacceptability of risk, we consider that more than 6 scores, but less than 12 scores is an acceptable risk with verification, if the level is less than 6 scores, the risk is acceptable, if more than 12 scores, it is unacceptable.

In the sixth step, actions to control general occupational and ergonomic risks are determined if the risk is considered acceptable during the inspection. The seventh step provides for the development of precautionary and protective measures to eliminate the risk or reduce its level to an acceptable level for general occupational ergonomic risk, the level of which is unacceptable. In the eighth step, we list all acceptable and permissible general occupational and ergonomic risks in the form of a map of occupational and ergonomic risks at the workplace. We also document unacceptable risks that will become acceptable if certain precautionary and protective measures are taken. Further, in the ninth step, enterprise managers or the responsible person must approve the register of occupational and ergonomic risks.

In the tenth step, according to the risk register, a plan for monitoring, eliminating and reducing occupational and ergonomic risks is developed and approved by the management of the enterprise (Table 10), taking into account the relevant deadlines, responsible persons and necessary resources.

**Table 10:** Form of plan for control, elimination and reduction of occupational and ergonomic risks.

No	Occupational and ergonomic risk is not acceptable	A measure to control, eliminate or reduce the risk	Execution period	Resources	Responsible person
1	Uncomfortable working posture	Anticipate the need to revise the technology of production operations, identify working positions with minimal ergonomic risk, provide ergonomic hand tools, and a procedure for automating production operations	Term from month to a half of a year	Making a change in the production technology involves financial costs within the developed budget	Production manager

In the last, the eleventh step, we anticipate the need to revise the register of occupational and ergonomic risks and the plan for monitoring, eliminating and reducing occupational and ergonomic risks at least once a year or in the event of significant changes in the work of an employee at the workplace in terms of introducing new equipment, recruiting workers of a different gender, etc. (41).

### 2.3. Collection and processing of data

To provide an example of determining occupational and ergonomic risk using the developed algorithm, a study of the workplace of a tree feller (logger) is conducted. This profession has been chosen because it is one of the most traumatic, which leads to disorders and diseases of the musculoskeletal system (42, 43). For example, hand vibration syndrome, carpal tunnel syndrome (44).

Based on identifying the causes of the development of the musculoskeletal system diseases among loggers, key indicators have been identified by which occupational and ergonomic risk can be assessed from published scientific literature (45–48). This list will also be refined based on surveys and observations, which are among the most common tools for identifying ergonomic risks (49, 50).

A test to determine the level of occupational and ergonomic risk of loggers was conducted at SE “Kievsky forestry” enterprise. The territory of SE “Kievsky forestry” is located in the central part of Kyiv Oblast, where broad-leaved forests (oak, hornbeam, ash, alder, and linden) predominate. The average age of loggers is 36 ± 3.1 years. The participants’ work experience ranges within 5 – 11 years. All participants participate in the research voluntarily. They were previously introduced to the testing program and control indicators, determined during the experiment.

The work of a logger involves felling trees, cutting branches, and sometimes it becomes necessary to clean the chainsaw, as well as other additional equipment, from dirt and wood debris; removing and washing the chain, cleaning the carburetor mesh and the fan; technical maintenance of the hydraulic felling wedge and other auxiliary tools. To fell, cut trees and trim branches, fellers use a Stihl MS 362 two-stroke mechanical chainsaw with a power of 3.4 kW and a weight of 5.9 kg.

**Figure 4:**
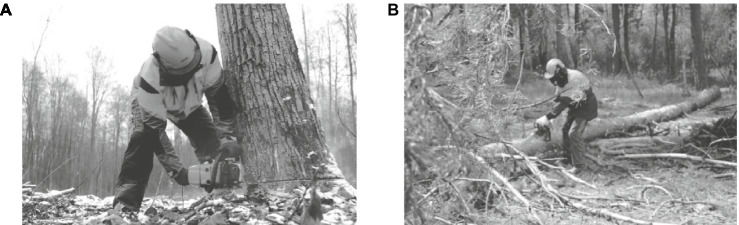
Basic production operations of the wood production technological process: **(A)** – felling trees; **(B)** – cleaning trees from branches and twigs.

To assess the working posture, a sample of photographs of production operations of felling trees, clearing trees of branches and twigs is used (Figure 4). To photograph the working postures of workers, a camera with a resolution of 1,024 × 768 pixels (Canon EOS R10 RF-S 18-45 IS STM) is used. The photographs were taken at a distance of one meter so that the lens captured the worker’s entire body in profile. All working movements of workers were photographed to determine ergonomic risk.

To set the pace and rhythm of work, when monitoring the performance of their work, count the number of movements per unit of time, determined by the task nature (felling trees or cutting branches). In this case, a scale from 1 to 5 is used, where 1 is not a high speed of work performance, and 5 is a very high speed of work performance (33, 51).

To assess the occupational and ergonomic risk of a logger, a team of five volunteer experts was formed with higher education in occupational safety and health. Experts have at least six years of experience working at forestry enterprises in the field of occupational safety. Each of the experts, after inspecting the logger’s workplace, observing his work and conducting a survey, is asked to determine the appropriate scores presented in Tables 6–8 regarding the probability of physical overload, the intensity of physical load and its duration. The scores set for each dangerous factor are averaged and entered into the appropriate table for further calculations.

The employee’s health indicators and adaptability index to overload have not been determined in this research for ethical reasons, but the proposed algorithm provides such an opportunity, which will make it possible in the future to select precautionary and protective measures taking into account the individual capabilities of the employee.

Score calculations and measurement discrepancy determinations are made using Microsoft Excel 2016. Outliers are tested using Z-scores, and all values are within ≤2, *p* < 0.05.

## 3. Research results

Based on field observations and a survey of loggers, dangerous factors characteristic of the SE “Kievsky forestry” enterprise have been determined. Table 11 shows the averaged data on the probability of physical overload, as well as the physical load intensity and its duration.

**Table 11:** Logger observation of dangerous ergonomic factors and occupational ergonomic risk components (employee questionnaire).

No	Group of dangerous factors	Dangerous factors		Physical overload			Physical overload consequences
		Name	Indication	Probability, Pr	Intensity, Int	Duration, D_pho_	
1.	Group of factors in the direction of joint movement, force magnitude, load level and activity state	Uncomfortable working posture: bent torso, outstretched arms, strain on the legs	DF_11_	2,6±0,4	4,6±03	4,7±0,3	Development of MSDs disease, muscle strain, tendon rupture, muscle pain, fatigue
		Working pace	DF_12_	2,7±0,3	3,2±0,3	4,8±0,4	
		Rhythm of work	DF_13_	2,6±0,4	2,6±0,3	2,8±0,3	
		Dynamic loads	DF_14_	4,2±0,5	3,8±0,5	4,2±0,3	Rapid loss of working capacity
2.	Group of dangerous factors – working environment	Air temperature	DF_21_	4,6±0,4	4,8±0,5	2,6±0,5	Overheating of the body
		Presence of wind	DF_22_	4,2±0,3	3,2±0,4	4,8±0,3	Fractures of limbs
		Fog	DF_23_	1,2±0,2	2,6±0,3	2,4±0,2	Fractures of limbs, spine
3.	Group of dangerous factors – equipment	Equipment weight	DF_31_	4,6±0,4	4,6±0,3	4,8±0,3	Loss of working capacity, muscle pain
		The number of repetitive movements	DF_32_	4,8±0,5	2,6±0,3	2,8±0,3	Loss of working capacity, muscle pain
4.	Index of employee’s adaptability to overload	Tension of adaptation mechanisms	*K_AD5_*	1.0			Possibility of physical health recovery
5.	Index of employee’s health condition	The level of employee’s individual health	*k_H5_*	1.0			
6.	Employee’s gender index	The employee's gender is male	*k_G1_*	1.0			

No group of dange-rous factors	Assessment of the residual level of occupational and ergonomic risks, R_i_	Precautionary and protective measures (PM) to reduce the unacceptable primary level of occupational and ergonomic risk, *Ri*	
		Indication	Content
1	AR	PM_11_	Review and implement new cutting technology, replace manual labor with automated systems, and provide for the possibility of increasing technological breaks.
	AR	PM_12_	
	AR	PM_13_	
	AR	PM_14_	
	**AR**		
2	AR	PM_21_	Conduct an assessment of climatic conditions, ensure control over the prohibition of work in unsuitable conditions, and provide for the possibility of performing other work.
	AR	PM_22_	
	AR	PM_23_	
	**AR**		
3	AR	PM_31_	Conduct an ergonomic assessment of chainsaws, select and use the most suitable one in terms of weight and other indicators.
	AR	PM_32_	Review and implement new cutting technology, replace manual labor with automated systems
	**AR**		
**ARC**			

It has been revealed that the most influential dangerous factors are the group that characterizes the direction of joint movement, the magnitude of effort, the level of load and state of activity. This includes directly an uncomfortable working posture, which arises due to the need to cut as low as possible at an angle to the trunk of 90°, a significant pace and rhythm of work (the tree must be felled as soon as possible, up to 320 s). Dynamic loads also occur when clearing a tree from branches positioned at different angles on the trunk (52). Also, when cutting trees, the terrain and weather conditions (snow, rain, wind, fog) influence the process (53). The results of processing scores from specified indicators, rounded for the convenience of their analysis and setting the risk level, are given in Table 12. Also, if an unacceptable level of risk is set, appropriate measures are provided to improve the logger’s safety. It should be noted that in order to maintain high labor productivity of the logger, preference is given to measures to change cutting technology, the use of various devices that reduce the number of dangerous working postures, as well as the elimination of equipment that increases the manifestation of negative health consequences.

**Table 12:** The form and example of a map for managing occupational and ergonomic risks of a logger.

No group of dange-rous factors	Dange-rous factors (DF)	Determination of the occupational and ergonomic risk initial level				Assessment of the occupational and ergonomic risk level, *R_i_*	
		Determination of severeness of consequences			Severeness of consequences, S_cons_, *S_cons_* = *D_pho_* × *I_nt_* × *k_AD_* × *k_H_* × *k_G_*/5	Occupational and ergonomic risk level, *R_i_* (abbreviation of words Figure 3)	
		Probability (frequency), *Pr*	Intensity, *Int*	Duration, *D_pho_*			
1	DF_11_	3	5	5	5	15	UNAR
	DF_12_	3	3	5	3	9	ARC
	DF_13_	3	2	5	2	6	AR
	DF_14_	2	5	4	4	8	ARC
	**Group 1 general risk**					**66**	**UNAR**
2	DF_21_	5	5	3	3	15	UNAR
	DF_22_	4	3	5	3	12	ARC
	DF_23_	1	5	2	2	2	AR
	**Group 2 general risk**					**29**	**UNAR**
3	DF_31_	5	1	5	5	25	UNAR
	DF_32_	5	5	3	3	15	UNAR
	**Group 3 general risk**					**115**	**UNAR**
**General risk of all groups**						**205**	**UNAR**

An analysis of the research results shows that the logger is exposed to several significant dangerous factors: an uncomfortable working posture, which, together with rather dangerous equipment, leads to the risk of developing the occupational MSDs. This requires, first of all, a reduction in the intensity and duration of the employee’s physical load, which will reduce the severity of the health consequences. Of course, it is necessary to consider the employee’s adaptability – recovery and the current state of his physical health (54). At the same time, constant exposure to dangerous factors from production equipment and the environment can reduce these opportunities due to the need to use a certain amount of energy to adapt to uncomfortable working conditions (55, 56).

## 4. Discussion

A distinctive peculiarity of the proposed approach to determining the level of occupational and ergonomic risk from the known ones is the calculation of the adaptability to physical loads and the state of health of the employee. On the one hand, thanks to this approach, it is possible to individually take into account the distribution of work, embodying the well-known TILE principle (Task, Individual, Load, and Environment) (57) to reduce MSDs. On the other hand, it can be used to provide employees with an appropriate level of workload, which will help avoid injuries to employees with low physical strength and musculoskeletal system chronic diseases.

From a methodological point of view, the proposed process allows for the assessment of occupational and ergonomic risk. That is, ergonomic risks associated with working posture, load, and equipment are assessed taking into account occupational hazards related to the organization of the production process, the impact of working conditions, etc. In addition, the proposed method takes into account the influence of the employee’s individual parameters, which makes it possible to make targeted management decisions on health preservation by ensuring that the workload corresponds to the health level. Therefore, this can be considered a progress over existing tools.

The proposed process begins with an analysis of the task, individual characteristics of the employee’s health, analysis of the characteristics of the production tools, working conditions, which makes it possible to clearly identify the danger and dangerous factors that lead to an increase in the probability of the dangerous event occurrence. Then appropriate methods are used to determine the occupational and ergonomic risk for each dangerous factor. By the way, these tools include, among other things, all known tools for ergonomic parameters, if they allow the evaluation scales to be combined. In this example, a 5-score scale was chosen, but it can be changed for convenience and detailing of assessments.

In general, the main difference between the implemented approach to managing occupational and ergonomic risks from the known ones is that it takes into account a number of dangerous factors: hygienic, psychophysiological and individual, which are absent in other available models. It is assumed that the greatest influence on musculoskeletal disorders is danger – especially inappropriate, uncomfortable, unnatural working posture (10, 12, 58). At the same time, it is not taken into account that the amount of risk may be further aggravated by psychosocial dangers (59) that arise from the organizational culture, psychological climate, environmental parameters, which are assessed according to hygienic principles. However, if to take into account the totality of various dangerous factors, it is possible to set this task much broader than identifying the causes of occupational MSDs. For example, as mentioned above, this may be an increase in labor productivity, taking into account the absence of injuries and the absence of occupational diseases, but is more attractive to business owners in terms of justifying changes in the technological process, production equipment, and hand tools.

The positive result of implementing this approach is the preservation of the organization’s value, primarily human potential, which is based on the appropriate and timely involvement of interested people and a structured, comprehensive approach to risk management. Hence, there is a need to develop basic principles for managing occupational and ergonomic risks. In particular, the revealed patterns between the amount of occupational and ergonomic risk and the influence of dangerous factors (intensity of joint movement, high magnitude of effort, overload and state of activity; working environment, equipment) allow us to speak about the development of a strategy aimed at eliminating problems related to the human factor. It is assumed that this will be a complex of various measures for mechanization and automation of the technological process to replace human manual labor. This requires awareness of the most important dangerous factors influencing decision-making, which entails conducting appropriate training to improve the competence of employees, thereby forming an appropriate leadership institute (60). Taken together, this will ensure a positive working environment where responsibility, motivation, training and development play an important role and are based on occupational ergonomic risk management practices.

Management of occupational and ergonomic risks is, first of all, work with people, which is related to the impact on their health of physical load, movements, working postures, organization of production, which is dictated by public interests. Therefore, to achieve real results in reducing the incidence of MSDs, it is necessary to involve employees in the process of managing occupational and ergonomic risks. It is important that employees are involved in this process to protect human health or preserve valuables (61). In the future, there is a need to optimize the organization’s activities due to the emergence of various tasks to ensure the reliability and effectiveness of the production process.

## 5. Conclusion

An algorithm for the process of managing occupational and ergonomic risk has been developed, consisting of eleven steps, which can be nominally divided into three steps: preparatory, main and documented. In the process of managing occupational and ergonomic risks, it is necessary to take into account the dangerous factors of physical overload that are related to work: the direction of joint movement, the magnitude of effort, the level of load and the state of activity; working environment, equipment (infrastructure); employee’s adaptability to physical overload; physical condition of health and gender of the employee.

It has been determined that occupational and ergonomic risk is the probability of the dangerous event occurrence due to physical overload of an employee and its impact on the severity of damage to the employee’s physical health.

The level of occupational and ergonomic risk in the process of managing occupational and ergonomic risks is determined taking into account the probability (frequency), intensity and duration of physical overload, as well as adaptability to physical overload, the health condition and the gender of the employee.

The principles of occupational and ergonomic risk management are substantiated. They are based on the bow-tie model and predict the impact on the probability and severity of the dangerous event consequences, taking into account dangerous factors: high intensity of joint movement, high magnitude of effort, overload and state of activity; working environment, equipment (infrastructure); employee’s adaptability to physical overload; physical health condition and gender of the employee.

Maps of occupational and ergonomic risks have been developed, in which it is necessary to take into account the interaction of occupational hazards, as well as occupational and ergonomic dangers – physical overload.

Two approaches to assessing occupational and ergonomic risk are proposed: the first takes into account the probability (frequency), intensity and duration of physical overload, and the second takes into account the employee’s adaptability to physical overload, index of health condition and gender.

## Data availability statement

The original contributions presented in the study are included in the article/supplementary material, further inquiries can be directed to the corresponding author.

## Author contributions

VT: conceptualization. SC and OD: methodology. OD and OBo: validation and resources. SC: formal analysis. VT, SC, and OD: investigation. VT and OBo: writing-original draft preparation. SC and VL: writing-review and editing. OD: visualization. VT and SC: supervision. OBa: project administration. OBa and VL: funding acquisition. All authors contributed to the article and approved the submitted version.
